# The effect of a number of H9C2 rat cardiomyocytes passage on repeatability of cytotoxicity study results

**DOI:** 10.1007/s10616-016-9957-2

**Published:** 2016-03-05

**Authors:** Piotr Witek, Agnieszka Korga, Franciszek Burdan, Marta Ostrowska, Beata Nosowska, Magdalena Iwan, Jarosław Dudka

**Affiliations:** 1 Independent Medical Biology Unit, Medical University of Lublin, Chodzki 6, 20-093 Lublin, Poland; 2 Department of Human Anatomy, Medical University of Lublin, Jaczewskiego 4, 20-090 Lublin, Poland

**Keywords:** Cytotoxicity, H9C2 cell line, Passage number

## Abstract

The embryonic cardiomyocyte cell line H9C2 is commonly used in numerous in vitro studies, including cardiotoxicity analyses of new drugs. So far no results were published for studies on cell parameters variability during the cell line ageing process. For this reason the aim of the study was to evaluate the effect of a number of H9C2 rat embryonic cardiomyocytes passages on repeatability of study results for selected cytotoxicity parameters, with doxorubicin as a model toxic agent. The cultures were passaged twenty-five times. Cells from passage 1, 5, 10, 15, 20 and 25 were treated with doxorubicin for 24 h. Then drug cytotoxicity was evaluated with the MTT test and additionally the nuclear factor erythroid 2–related factor (Nrf2) gene expression was examined. The analysis of oxidative stress intensity and cell morphology was also assessed. The microscopic appearance of cells indicates that untreated cardiomyocytes morphology changes as well as sensitivity to toxic effects increases with the number of passages. Also an increase in oxidative stress in cells occurs with further passaging of cardiomyocytes. Statistical significance of differences in conducted tests results depended on doxorubicin concentration but in many cases the H9C2 line was found to be a reliable in vitro model only for the first five passages. For this reason it is important to take into consideration that further culturing of cardiomyocytes may not ensure repeatability of study results due to the culture ageing.

## Introduction

The H9C2 line of embryonic rat cardiomyocytes is a subclonal line of the original clonal cell line derived from embryonic BD1X rat heart tissue by Kimes and Brandt (1976). This line is commonly used in numerous in vitro studies because morphological parameters of their cells resemble immature embryonic cardiomyocytes (Louch et al. 2001; Tan et al. 2010; Watkins et al. 2011). However, these cells retain some components of the signalling pathway essential for their differentiation into mature cardiac muscle cells. The cell line is used, in particular, for cardiotoxicity analyses of new, mainly anticancer drugs (e.g. doxorubicin), and for studies on mechanisms of myocyte damage, and assessment of toxic effects of studied compounds on apoptosis and necrosis in cardiac myocytes. Embryonic H9C2 cardiomyocytes proliferate well in in vitro conditions, allowing relatively easy culturing (Louch et al. 2001; Watkins et al. 2011; Fu et al. 2005). However, contrary to immortalized cell lines, cardiomyocytes can be cultured for limited number of passages. Cell parameters variability during the ageing process may be of significance for repeatability of study results (Fu et al. 2005).

Despite common use of this line, so far no results were published for studies on cell ageing rate. Therefore, the aim of this study was to evaluate the effect of a number of H9C2 rat embryonic cardiomyocyte passages on repeatability of study results for selected cytotoxicity parameters, with doxorubicin as a model toxic agent against cardiomyocytes.

## Materials and methods

### Cell culture

The study was conducted on an adherent H9C2 line of rat embryonic cardiomyocytes (ATCC, Manassas, VA, USA). Cultures were grown on Dulbecco’s Modified Eagle Medium (DMEM, PAA Laboratories, Pasching, Austria), supplemented with 10 % foetal bovine serum (Life Technologies, Carlsbad, CA, USA). Cells were cultured at 37 °C in 5 % CO_2_-air. The cultures were passaged after 70–80 % confluence was achieved. Cells were rinsed with PBS solution, and then released with trypsin and EDTA solution (Life Technologies). The suspension of released cells was centrifuged at 120×*g* for 5 min. Cardiomyocytes were inoculated into a new culture bottle in concentration of 2 × 10^4^ cells/ml. 25 passages were made in total. Cells from passages 1, 5, 10, 15, 20 and 25 were treated with doxorubicin (DOX, EBEWE Pharma, Unterach, Austria), at 0.5, 1, and 5 µM, for 24 h, each cytotoxicity experiment was repeated five times.

### Cell morphology analysis

Cell morphology was analysed under a phase-contrast microscope Nikon Eclipse Ti. In each evaluated culture an area of minimum 20 cell nuclei was measured using NIS-Elements Imaging Softwere (Nikon, Tokyo, Japan) and statistically analysed.

### Cytotoxicity analyses

Doxorubicin cytotoxicity was evaluated with the MTT (3-(4,5-dimethylthiazol-2-yl)-2,5-diphenyltetrazolium bromide) test, using the MTT Cell Proliferation Assay Kit (Life Technologies). The test principle is based on live cells ability to reduce orange tetrazolium salt to water-insoluble purple formazan crystals. MTT solution (4 mg/ml) was added to the culture 24 h after doxorubicin. Following 4 h incubation, the medium with MTT was removed, and the formed crystals were dissolved in DMSO. The solution absorbency was measured at 540 nm, using PowerWave™ microplate spectrophotometer (Bio-Tek Instruments, Winooski, VT, USA). The measurements were performed in triplicate.

### Nrf2 mRNA expression

Total RNA was extracted with TRIzol reagent (Life Technologies) according to the manufacturer’s protocol. cDNA was synthesized using High Capacity cDNA Reverse Transcription Kit (Life Technologies) following the manufacturer’s instructions. Quantitative real-time PCR was performed in triplicate by using TaqMan^®^ Gene Expression Assays: Nfe2—Rn00477784_m1 and 18S—Hs99999901_s1 (Life Technologies) on 7500 Fast Real-Time PCR system (Life Technologies). Relative gene expression levels of Nrf2 were determined using ΔΔCt method and presented as RQ value (RQ = 2^−ΔΔCt^). As reference 18S gene was used.

### Oxidative stress

Oxidative stress was evaluated under a fluorescence microscope, using Mito-Tracker Green FM and RedoxSensor Red CC-1 (Life Technologies). Mito-Tracker Green FM passively diffuses through the cell membrane and accumulates in active mitochondria, resulting in green fluorescence. Location of Redox Sensor Red CC-1 marker depends on the cytosolic redox potential of the cell—the marker can be oxidised in cytoplasm and accumulates in mitochondria, resulting in red fluorescence, or is transported to lysosomes and there it is oxidised, emitting a fluorescence signal. Cells were incubated with staining agents at 37 °C for 10 min. After incubation, cells were rinsed twice with the PBS solution and observed under the Nikon Eclipse Ti microscope. Stains colocalisation in mitochondria was evaluated on photographs taken in two colour channels and superimposed in NIS-Elements Imaging Software.

### Statistical analysis

The results obtained for MTT test, gene expression analysis and cell nuclei area measurement were analysed statistically in the STATISTICA v. 8.0 application (StaftSoft, Cracow,Poland), using mean and standard deviation values. To compare more than two groups, the one-way analysis of variance ANOVA and post hoc multiple comparisons on a basis of Tukey’s HSD test were used.

## Results

### Changes in cell morphology

**Fig. 1:**
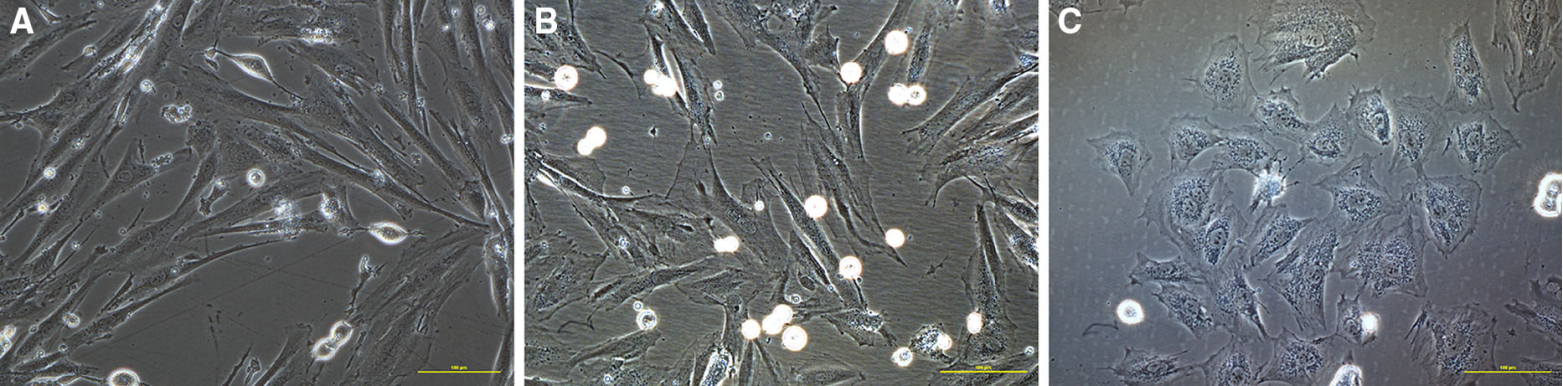
Control cardiomyocyte H9C2 **a** passage I, **b** passage X, **c** passage XXV

**Fig. 2:**
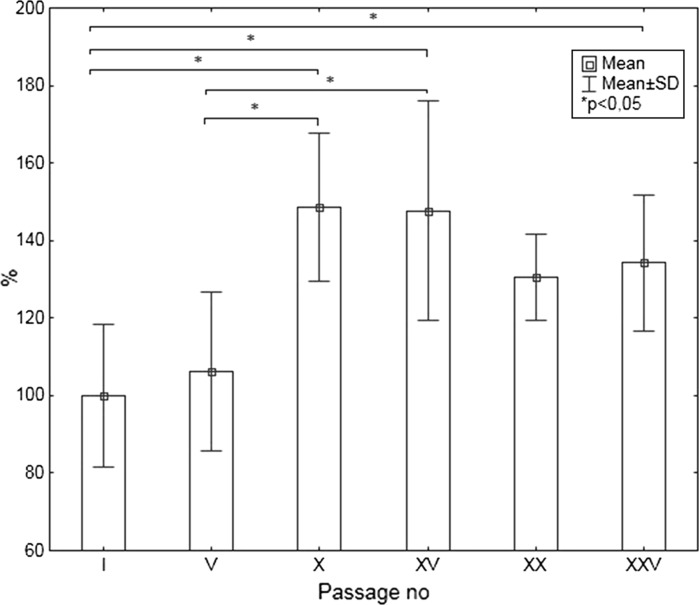
Untreated cells nuclei area. Data are presented as % of passage I control cell nuclei size

After the first passage spindle-shaped mono- or polynuclear cells were visible. After the fifth passage cells looked comparable but from passage X cells had characteristic enlarged nuclei and tended to change its shape from spindle to ameboid. Furthermore, varying intensity of granules aggregation around the nuclei was observed (Fig. 1). Statistical analysis of nuclei area showed significant differences beginning with X passage (Fig. 2).

**Fig. 3:**
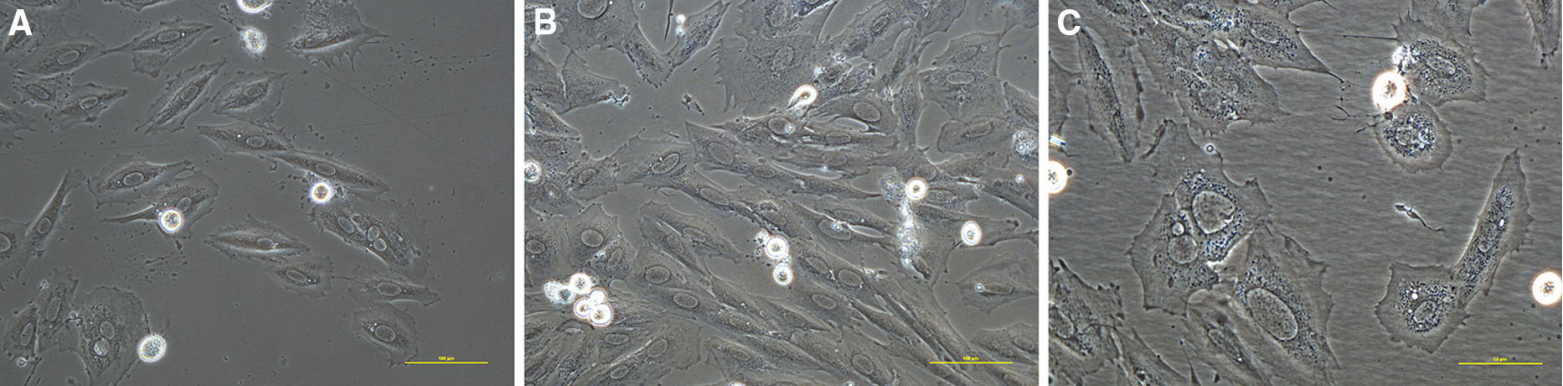
Cardiomyocyte H9C2 treated with 5 µM DOX. **a** passage I, **b** passage V, **c** passage XXV

**Fig. 4:**
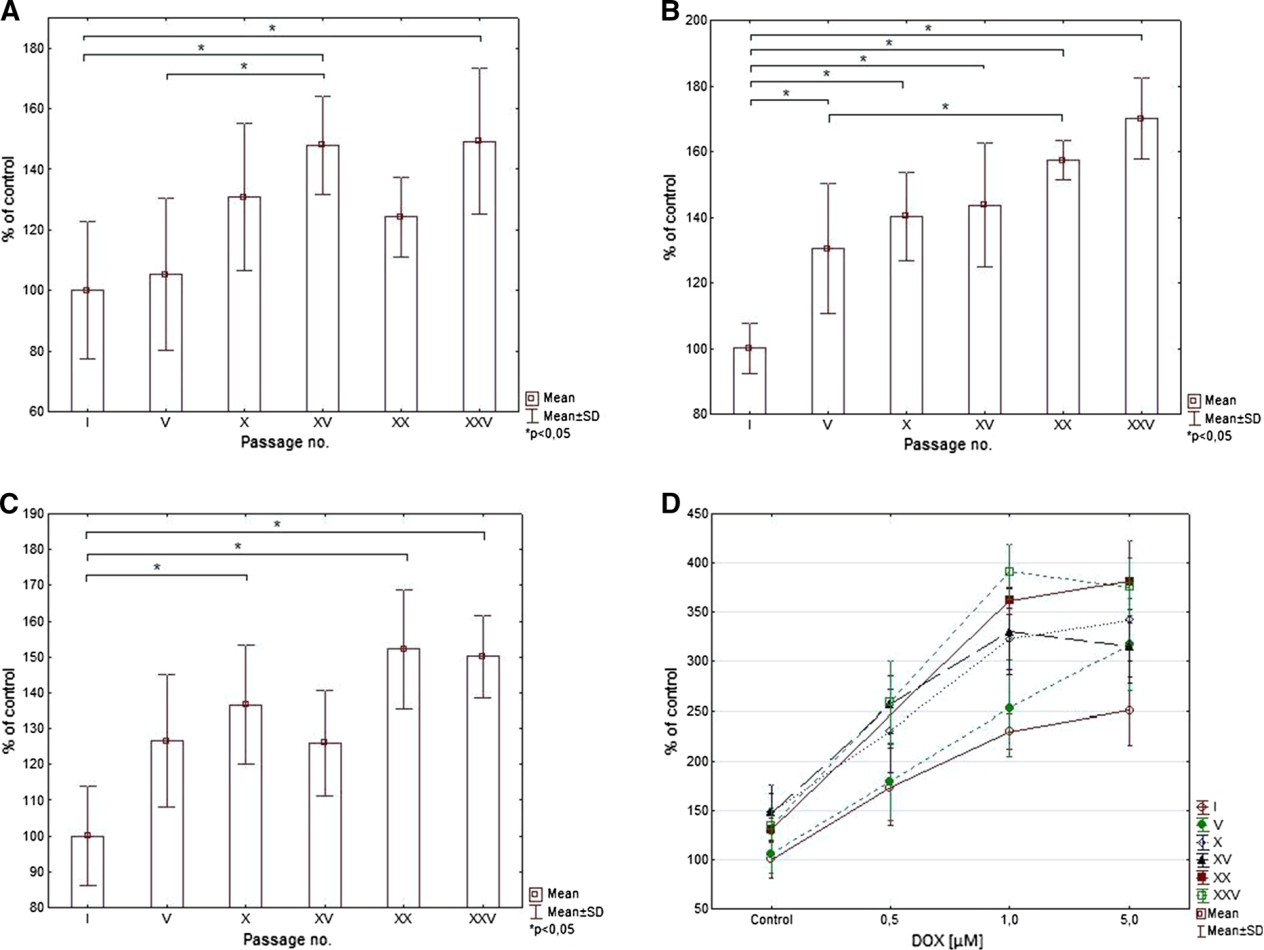
The nuclei area of cells treated with DOX according to passage number for **a** 0.5 µM, **b** 1 µM, **c** 5.0 µM of DOX. Data expressed as % of I passage cell nuclei size. **d** Analysis of nuclei area of cells in different passages according to DOX concentration. Data expressed as % of I passage control cell nuclei size

Described morphological changes specific for aging cells (enlarged nuclei, ameboid shape and presence of granules) were also noticed in cells treated with DOX (Fig. 3). The changes became more intensive in the subsequent passages, in particular for the highest concentration of DOX. We noticed that statistically significant changes in volume of nuclei start from passage V, X or XV in dependence of the concentration of the antibiotic (Fig. 4).

### MTT test

**Fig. 5:**
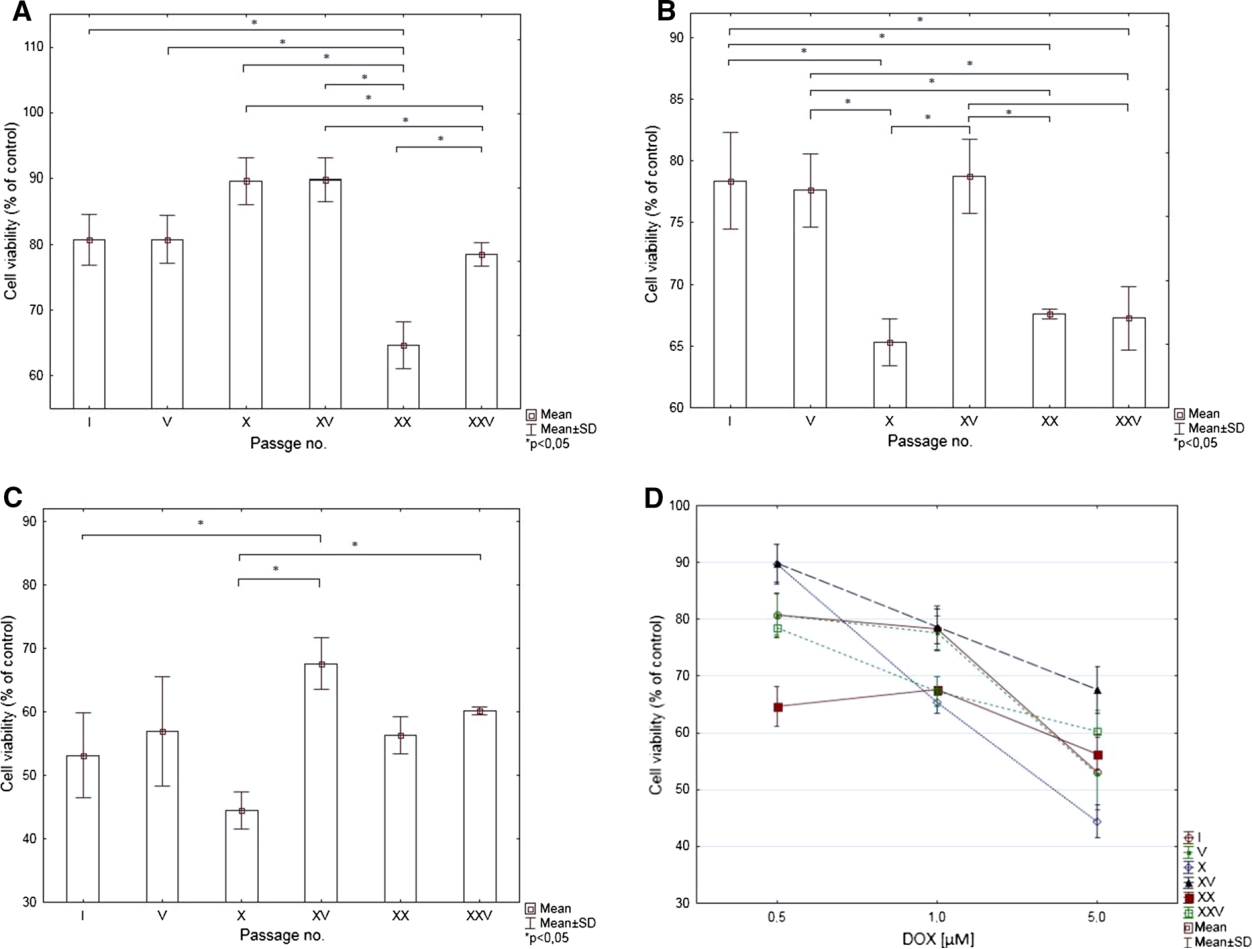
MTT test results for cardiomyocytes treated with DOX expressed as % of values obtained for control cardiomyocytes according to passage number for **a** 0.5 µM, **b** 1 µM, **c** 5.0 µM of DOX. **d** MTT test results for cardiomyocytes in different passages according to DOX concentration. Data are expressed as % of values obtained for control cardiomyocytes

Differences in MTT test results strongly depended on toxic agent concentration. Interestingly the smallest variability in results between successive passages was observed for the highest concentration of DOX. It can also be observed that curve depicts the relationship between viability versus DOX concentration have different shape for I and V passage in comparison to the subsequent passages (Fig. 5).

### Nfr2 mRNA expression

**Fig. 6:**
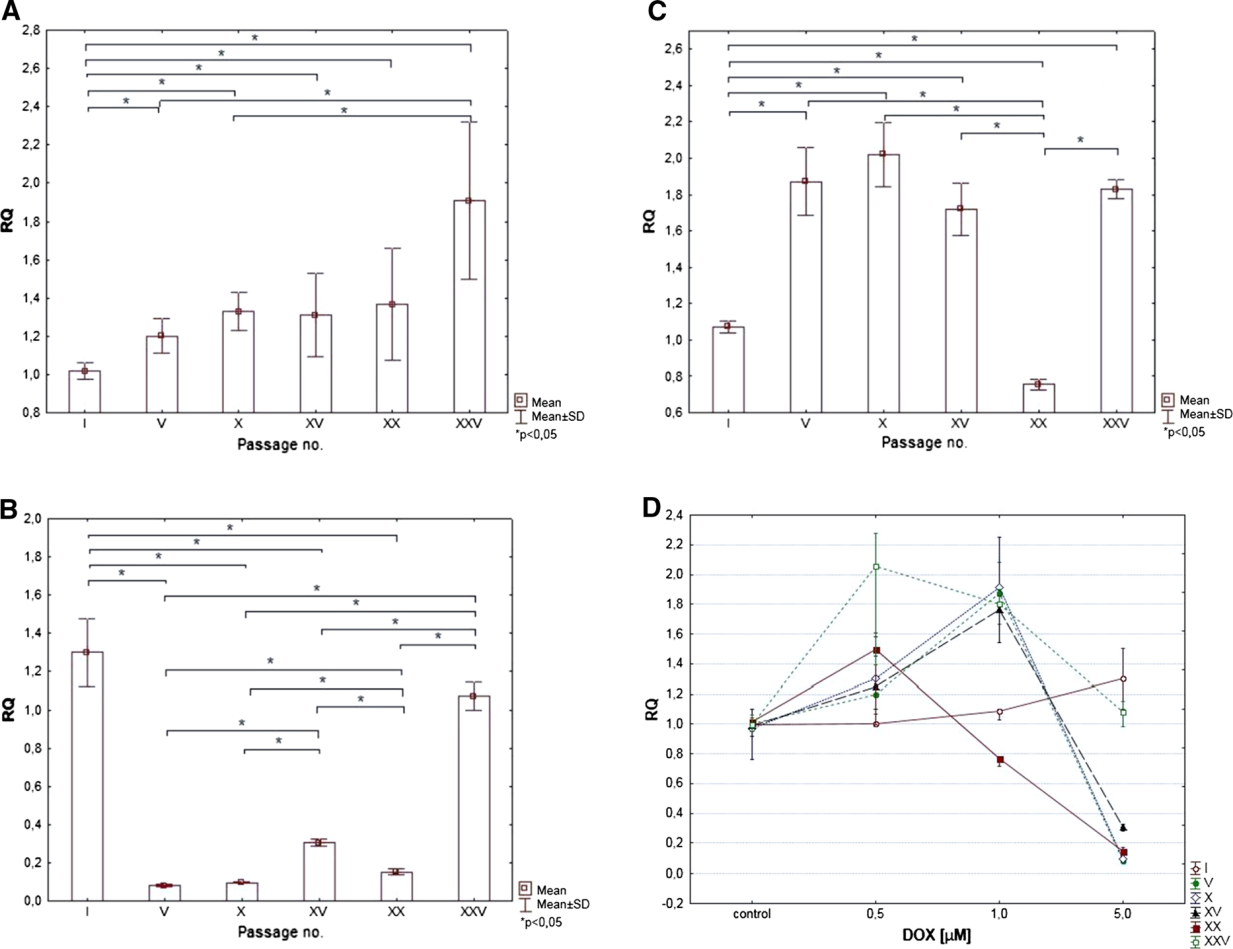
mRNA expression of Nrf2 gene in cells treated with DOX according to passage number for **a** 0.5 µM, **b** 1 µM, **c** 5.0 µM of DOX. **d** mRNA expression of Nrf2 gene for cardiomyocytes in different passages according to DOX concentration Data are expressed as RQ value

The lack of reproducibility in studied mRNA expression for a gene *Nfr2* between cells from the first and remaining passages was noticed. Expression of the gene tended to increase with cell aging in the case of cells treated with 0.5 and 1 µM DOX (expect XX passage). The highest DOX concentration caused significant decrease of Nfr2 mRNA expression in V, X, XV and XX passage (Fig. 6).

### Oxidative stress analysis

**Fig. 7:**
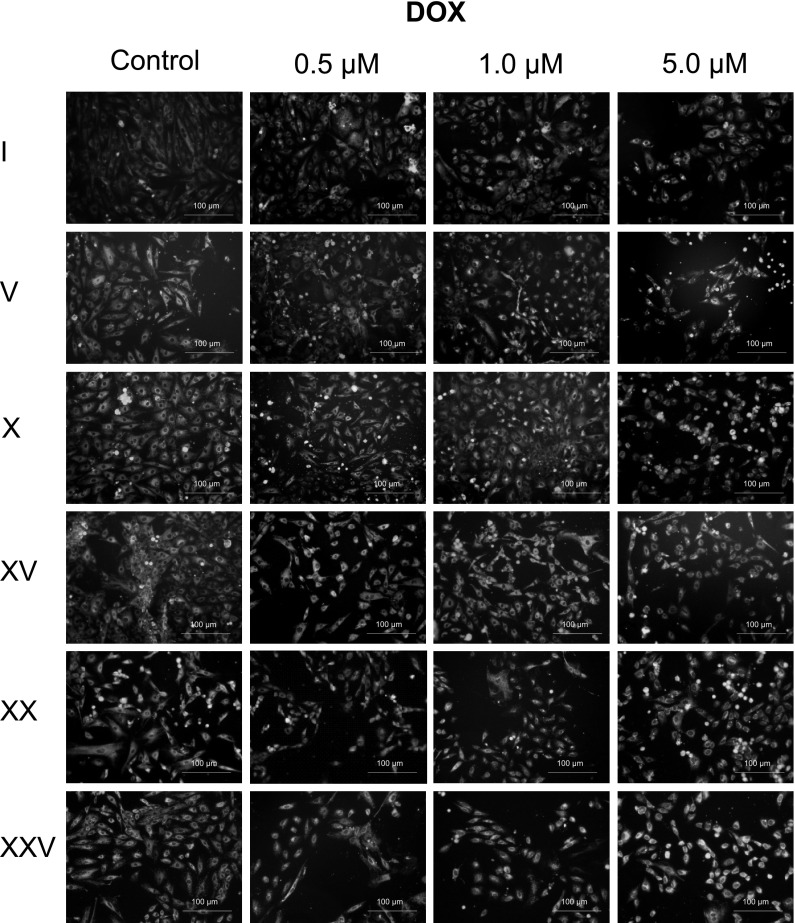
Oxidative stress intensity based on Mito-Tracker Green FM and RedoxSensor Red CC-1 colocalisation in mitochondria

In controls, and in cultures with DOX added at a relevant concentration (0.5, 1, 5 µM), oxidative stress is only slightly intensified in initial passages. With successive passages, increase in oxidative stress in cells in control cultures is observed. In passages with relevant doxorubicin doses added, the yellow signal is visibly intensified. At the same time, an increase in oxidative stress in cells occurs with further passaging of cardiomyocytes. The strongest intensification of the yellow signal is observed in passages 20 and 25, particularly for higher antibiotic concentrations (Fig. 7).

## Discussion

Cell ageing in non-immortalised cultures of cell lines used for in vitro studies is a natural and continuously progressing process. With the increasing number of cell line passages, changes in cell properties can be observed. As cells are an integral part of the study, they can affect results of experiments conducted on them. Determining the number of passages in which a given cell line is stable and test results remain repeatable seems to form a basis for any attempted studies. This procedure is of extreme importance in preclinical tests of new drugs. One of the lines used in tests of new drugs is the H9C2 line of rat embryonic cardiomyocytes. Its parameters are particularly useful in preclinical tests of anticancer drugs, determining their cardiotoxicity, safety, and possibility of moving to subsequent stages of clinical tests. Drug effects on cardiomyocytes are evaluated by determining cytotoxicity of a given compound, changes in cardiomyocyte morphology, percentage of necrotic cells, and the effect on proliferation (Tan et al. 2010).

Therefore, in this study an attempt was made to estimate the effect of the number of H9C2 line passages on repeatability of toxicological study results. Changes in cardiomyocyte response to a toxic compound (sensitivity to stress conditions) along with the age of the cell line were studied, together with progress in morphological changes in successive control passages. Cell morphology evaluated in microscopic observations indicated that the number of cells with changed morphological parameters increased in controls in which the antibiotic was not used. Use of a factor inducing structural changes in cells, such as DOX, only intensified these changes, and this was particularly visible in final passages. As data in the literature indicate, effects of this anthracycline on cells include generation of reactive oxygen and nitrogen species, thus inducing oxidative stress (Chen et al. 2015; Dudka et al. 2012; Narumi et al. 2015; Zhang et al. 2009). The intensified oxidative stress visualised in last passages with relevant fluorescent stains may be caused by increased mitochondrial dysfunction in cardiomyocytes. The test results in first passages indicated that young cells endured the DOX effects relatively well. This seems to be confirmed by Nrf2 gene expression analysis. This gene encodes a transcription factor that regulates the expression of genes containing the Antioxidant Response Elements (ARE) in their promoters. Nrf2 activates the expression of a range of cytoprotective proteins in response to toxic agents (Nguyen et al. 2009). In our study lack of reproducibility of mRNA expression was observed. After the first passage Nrf2 expression was slightly increased in the case of the highest concentration of DOX. In the following passages an increased expression at lower concentrations of the antibiotic and a decrease of gene expression at the highest concentration were observed, that may indicate reduced cell capability to defense (Bruns et al. 2015).

There was observed lack of reproducibility in MTT test result in subsequent passages. Interestingly, there was no particular trend in changes of cell viability based on this test. Although the MTT assay results generally correlate with the number of viable cells growing in standard culture conditions, the rate of tetrazolium reduction reflects the general metabolic activity or the rate of glycolytic NADH production (Berridge et al. 2005). There is possibility that cells of different passages are characterized by changed enzymatic activity without an effect on cell number or cell viability.

Publications concerning specification of standards for in vitro studies and the number of acceptable passages in specific cell lines are scarce. At the same time, there are no documented data available for passaging of cardiomyocytes. Kato et al. (2008) published results for the cultured human endometrial cancer line (CHEC), showing that with successive passages, cell morphology changes, together with expression levels for proteins such as cytokeratin, alpha-actin, or vimentin. Publications for other cancer cell lines include studies on the epithelial colorectal adenocarcinoma line, Caco-2, in which the changes in cell morphology occurring in successive passages were described, and an attempt was made to describe standards for culturing these cells to achieve repeatable results (Jahn et al. 2011; Natoli et al. 2012). In available literature, reports on studies on cultures derived from explants are scarce. Mazzocca at al., studying cells isolated from specimens of human tendons, proved that only freshly isolated human tenocytes can provide a reliable in vitro model solely in initial passages (only up to the first 2 passages) (Mazzocca et al. 2012). Studies conducted by O’Driscoll in stably-transformed mouse insulinoma cells MIN-6 showed significant differences in expression of mRNA responsible for secretion, adhesion and proliferation. Differences in expression of nearly thousand genes were demonstrated between cells from initial passages and cells from passage 40 (O’Driscoll et al. 2006).

The literature data and authors’ results indicate that the effect of cell line passage number on reliability and repeatability of study results is complex and depends on many factors, including the line type, tissue type, species, culture conditions, and, what is very important, applications for which they were used. Therefore, it is not possible to establish a universal method for determining the maximum number of passages for in vitro cultures.

## Conclusions

The studies conducted on the H9C2 rat embryonic cardiomyocytes indicate the cell response to toxic agents vary depending on the number of cell passages. Subsequent passaging leads to increased cellular sensitivity to stress conditions, and thus, to variable observed effects of a toxic agent.

Statistical significance of differences in conducted simple toxicological tests results depended on doxorubicin concentration but in many cases the H9C2 line was found to be a reliable in vitro model only for the first five passages. For this reason it is important to take into consideration that further culturing of cardiomyocytes may not ensure repeatability of study results due to the culture ageing. The effect of the number of passages is of significance for the toxicological tests reliability, and should be considered in preclinical tests of new drugs, and established each time individually for every experiment.
